# Impacts and Correlations on Corneal Biomechanics, Corneal Optical Density and Intraocular Pressure after Cataract Surgery

**DOI:** 10.3390/diagnostics14141557

**Published:** 2024-07-18

**Authors:** Fang-Yang Lin, Ren-Wen Ho, Hun-Ju Yu, I-Hui Yang, Po-Chiung Fang, Ming-Tse Kuo

**Affiliations:** 1 Medical Education Department, Kaohsiung Chang Gung Memorial Hospital, Kaohsiung City 83301, Taiwan; k64202@cgmh.org.tw; 2 Department of Ophthalmology, Kaohsiung Chang Gung Memorial Hospital, College of Medicine, Chang Gung University, Kaohsiung City 83301, Taiwan; wen6530@cgmh.org.tw (R.-W.H.); angelayu@cgmh.org.tw (H.-J.Y.); rubyihy@cgmh.org.tw (I.-H.Y.); fangpc@cgmh.org.tw (P.-C.F.); 3 School of Medicine, College of Medicine, National Sun Yat-sen University, Kaohsiung City 80424, Taiwan; 4 School of Medicine, Chang Gung University, Taoyuan City 33302, Taiwan

**Keywords:** corneal biomechanics, cataract surgery, corneal optical density, reversibility

## Abstract

The study aimed to investigate the extended effects and interrelations of corneal biomechanics, corneal optical density (COD), corneal thickness (CT), and intraocular pressure (IOP) following cataract surgery. Sixteen eyes were analyzed prospectively. The Corneal Visualization Scheimpflug Technology (Corvis ST) device assessed corneal biomechanics, while the Pentacam AxL^®^ (Pentacam) measured COD and CT. Postoperative data were collected around six months after surgery, with a subgroup analysis of data at nine months. The Pearson correlation was used to examine the relationship between surgical-induced changes in corneal biomechanics and COD. At six months, significant postoperative differences were observed in various biomechanical indices, including uncorrected IOP (IOPuct) and biomechanics-corrected IOP (bIOP). However, many indices lost statistical significance by the nine-month mark, suggesting the reversibility of postoperative corneal changes. Postoperative COD increased at the anterior layer of the 2−6 mm annulus and incision site. The changes in COD correlated with certain biomechanical indices, including maximal (Max) deformative amplitude (DA) and stiffness parameter (SP). In conclusion, despite significant immediate postoperative changes, corneal biomechanics, COD, and IOP experienced a gradual recovery process following cataract surgery. Clinicians should maintain vigilance for any unusual changes during the short-term observation period to detect abnormalities early.

## 1. Introduction

Corneal biomechanics has received great attention in the past two decades. It plays a crucial role in maintaining shape and viscoelasticity [1]. Diseases, interventions, or surgeries involving the cornea will affect corneal biomechanics more or less [2]. Although keratoconus and laser refractive surgery are the two most discussed topics [3,4], the impact of cataract surgery may not be underestimated.

Cataract surgery is, to date, the most common ocular surgery proven to be an effective and safe way to restore clear vision, enhancing the quality of life for those affected [5]. For less surgical manipulation and faster visual rehabilitation, clear corneal incision maintains the mainstream surgical approach in phacoemulsification cataract surgery [6,7], resulting in biomechanics change. 

The Corneal Visualization Scheimpflug Technology (Corvis ST; Oculus, Wetzlar, Germany) device is currently the most widely used method for measuring corneal biomechanics. A few studies demonstrated the effects on corneal biomechanics using the Corvis ST after cataract surgery [8,9,10]. One concluded that a less stiff cornea postoperatively led to falsely reduced intraocular pressure (IOP) measurements [10]. However, their results were limited by a short follow-up duration [8,9,10]. 

Endothelial damage is another unwanted but unavoidable complication during cataract surgery. Surgical errors or ultrasound energy during phacoemulsification can cause massive endothelial damage [11]. There are several tools to evaluate the condition of endothelium. Tools like confocal microscope and specular microscope directly quantify the number of endothelial cells, which were reported to reduce after cataract surgery [11,12]. Moreover, corneal optical density (COD), corneal thickness (CT), and corneal volume reflect the quality and function of the endothelium. All of them increased after cataract surgery [13,14].

Corneal biomechanics play an important role in shaping the cornea, and COD determines its transparency. Both elements are essential for the refraction of light–the main function of the cornea. Although they have been well studied, the relationships between corneal biomechanics and COD are still controversial. Previous research found a correlation between optical transparency and biomechanics measured with another corneal biomechanical analyzer, the Ocular Response Analyzer (ORA; Reichert Ophthalmic Instruments, Buffalo, NY, USA), in young healthy Caucasian students [15] instead of subjects with cataracts [16]. In keratoconus patients, the corneal densitometry values correlated with the SP of the Corvis ST [17]. Concerning the viscoelastic changes following cataract surgery, we hypothesize that COD is one of the factors that affect corneal biomechanics.

The study aimed to investigate the extended postoperative changes on corneal biomechanics, COD, and CT and to explore whether these effects related to cataract surgery are reversible and correlated to each other.

## 2. Materials and Methods

### 2.1. Participants

This prospective study was conducted from October 2020 to September 2023 at the Department of Ophthalmology in Kaohsiung Chang Gung Memorial Hospital. All works were completed in accordance with the Declaration of Helsinki and approved by the Institutional Board Committee (IRB) of Chang Gung Memorial Hospital (IRB certification no. 202001104B0). Inclusion criteria were cataract patients that meet surgical indications. Exclusion criteria included glaucoma, traumatic cataract, congenital cataract, active corneal infection, or previous ocular surgeries. 

### 2.2. Surgical Procedures of Cataract Surgery 

The same experienced ophthalmologist performed the cataract surgery with the standard procedures of phacoemulsification cataract extraction and posterior chamber intraocular lens (IOL) implantation. The surgical steps included a 2.75 mm clear corneal incision at the temporal site, ophthalmic viscoelastic device (OVD) use, manual continuous curvilinear capsulorhexis (CCC), divide-and-conquer technique for nucleus removal, and implantation of IOL. All subjects underwent uneventful surgery under topical anesthesia, and no surgical-related complication was observed intraoperatively or postoperatively.

### 2.3. Clinical Assessment 

Preoperative assessments were conducted one month before the cataract surgery, consisting of bare visual acuity (VA), keratometry, corneal biomechanics, COD, and CT. We followed the patients at the outpatient department one day, one week, and one month after the surgery, continuing with three-month intervals. Postoperative data were collected after at least three months of observation. 

**Figure 1:**
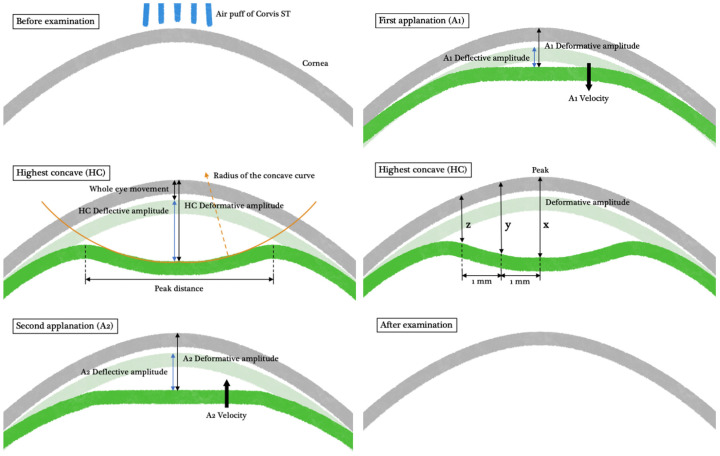
The deformative process of the cornea under Corvis ST examination. DA_c is the value of DA after correcting eye movement. DAR_1 mm is defined as x/y, while DAR_2 mm is x/z. Abbreviation: DA_c, deflective amplitude; DA, deformative amplitude; DAR_1 mm (DAR_2 mm), DA ratio between cornea apex and paracentral 1 mm (DAR_2 mm).

The Corvis ST is a non-contact corneal biomechanical analyzer providing a direct assessment of dynamic corneal response (DCR) [18]. The Corvis ST consists of an air puffer, producing a fixed-pressure air jet to deform the cornea, and an ultra-high-speed camera to capture the deformation process with serial images. Tonometry and pachymetry can also be visualized with great precision based on the Scheimpflug images. The Corvis ST with software version 1.6r2031 was adopted in our study. We defined the parameters that can be directly measured from the serial images as “Basic Parameters”, which were further divided into the indices at the first applanation (A1), the second applanation (A2), highest concavity (HC), and of limit (Figure 1). On the other hand, the parameters that required further calculation with basic parameters and patient-specific characteristics were referred to as “Integrated Parameters”, including the latest released stress-strain index (SSI).

**Figure 2:**
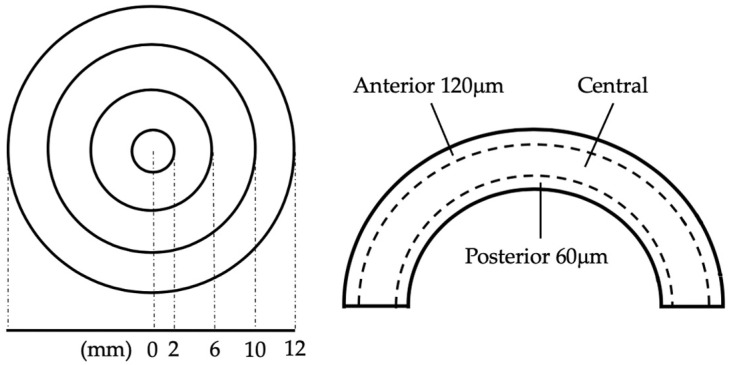
Measurements of COD. The cornea is divided into concentric annuli of 2, 6, 10, and 12 mm in diameter (**Left**), and in three different depths (**Right**) to measure for COD in corneal densitometry.

The Pentacam AxL^®^ (Pentacam; Oculus, Wetzlar, Germany) is a versatile machine for corneal densitometry and optical pachymetry [19]. We extracted COD and CT to assess the quality of the cornea. Corneal densitometry quantified the backscattering light from the exact position within the cornea as its COD. The whole corneal was divided into four concentric annuli centered on the thinnest point of the cornea (0–2 mm/2–6 mm/6–10 mm/10–12 mm in diameter) and three layers (anterior 120 μm/central/posterior 60 μm) for COD assessment (Figure 2) [19]. For a closer look at the clear cornea incision wound, we averaged the two COD values at the temporal-peripheral site of the densitometric map to represent the incision site [14]. CT was measured with corneal tomography, which reconstructed the three-dimensional cornea and created a pachymetric map after assessing its anterior and posterior surface [20]. CT was also evaluated in concentric annuli. The annuli had diameters ranging from 0.0 mm to 12.0 mm, with increments of 0.4 mm per annulus, depicting a spatial profile of CT.

### 2.4. Sample Size Estimation

The sample size was determined by G*Power (Germany, version 3.1.9.6). The estimation adopted the significance level (α) as 0.05 and the desired power (1 − β) as 0.8. The calculated effect size was −1.065 according to the result of biomechanics-corrected IOP (bIOP) from our 16 qualified patients. The estimated sample size for this study was at least 10 subjects.

### 2.5. Statistical Analysis

The Shapiro–Wilk normality test was performed first and two-tailed paired t-tests or Wilcoxon signed-rank tests were conducted according to their distribution to assess the change in preoperative and postoperative data. The Pearson correlation or Spearman correlation coefficient was further calculated to evaluate the association between corneal biomechanics and COD. Statistical analysis was performed with the online calculator Social Science Statistics “https://www.socscistatistics.com (accessed on 10 September 2023)” and Excel 2019. A significant level was set as a *p*-value < 0.05.

## 3. Results

### 3.1. Demographic Data

The original data collection enrolled information from 19 eyes of 16 patients. After data verification, three eyes were excluded due to insufficient follow-up duration and measurement error of the Corvis ST. The final assessment was conducted with 16 eyes of 13 patients, including 9 males (56.3%) and 7 females. The baseline demographic data are shown in Table 1. The mean age of the patients was 66.3 years old when receiving cataract surgery. In the study, 43.8% of the surgeries were performed on the right eye, and 56.3% on the left eye. The mean postoperative follow-up period was 5.81 ± 4.36 months. The average bare VA showed significant improvement postoperatively (*p* < 0.001).

### 3.2. Corneal Biomechanics

#### 3.2.1. Basic Parameters of the Corvis ST

After receiving cataract surgery, uncorrected IOP (IOPuct) decreased by 2.69 ± 2.52 mmHg (*p* < 0.001), whereas central corneal thickness (CCT) did not reach a statistical difference (Table 2). Among the A1 indices, A1 Time decreased by 0.517 ms (*p* < 0.001) and A1 Velocity accelerated by 0.024 m/s (*p* = 0.001). Only A2 Velocity remarkably increased (*p* < 0.001) among the A2 indices. In the group of the limit indices, postoperative maximal (Max) deformative amplitude (DA) and max deflective amplitude (DA_c) increased by 0.141 mm and 0.125 mm, respectively (*p* < 0.001). Significantly expanded peak distance and decreasing radius (*p* = 0.03) indicated a larger and deeper corneal concave when confronting the air puff of the Corvis ST.

#### 3.2.2. Integrated Parameters of Corvis ST

bIOP demonstrated a significant reduction by 2.39 ± 2.32 mmHg within a 6-month observation period (Table 2). Stiffness parameter (SP) at A1 (SP A1), SSI, Ambrosio Relational thickness horizontal (ARTh), and Integrated Radius changed after cataract surgery with statistical significance. The ratio between corneal apex and paracentral 2 mm (Max DAR_2 mm) also displayed a notable difference, while the ratio at 1 mm (Max DAR_1 mm) did not. The corneal biomechanical index (CBI), a combination index of DAR, Integrated Radius, ARTh, and SP-A1, showed no significant difference postoperatively (*p* = 0.16).

#### 3.2.3. Biomechanics Changes during Extended Observation

**Figure 3:**
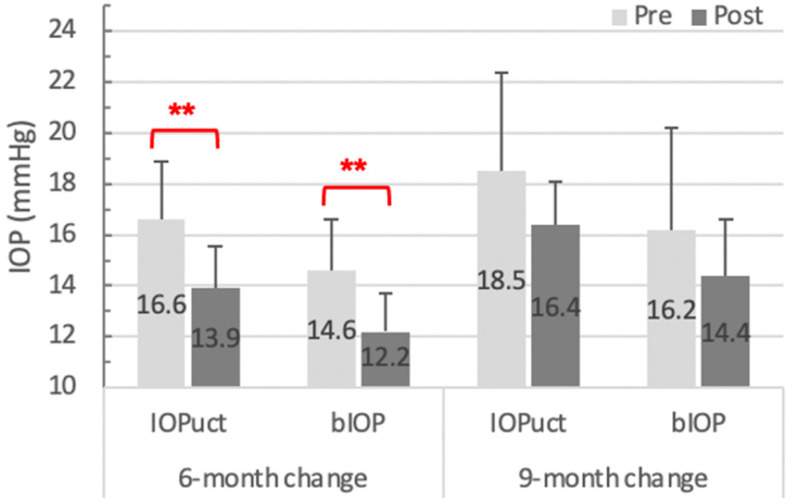
IOP changes in different follow-up periods. The 9-month change of both IOPuct and bIOP lost significance (*p* = 0.09, *p* = 0.12, respectively) when compared with the 6-month change (both *p* < 0.001). Significant differences defined as p < 0.05 were highlighted with **. Abbreviation: IOP, intraocular pressure; IOPuct, uncorrected IOP; bIOP, biomechanics-corrected IOP.

Seven patients with an average 9-month (9.29 ± 4.72 months) follow-up period were analyzed as an extended subgroup (Table 3). All basic indices that exhibited significant changes at the 6-month period lost their significance when observed up to 9 months, except for DA, DA_c, deflective area (Darea) at HC, DA_c, and peak distance at of limit. The statistical significance of ARTh, SSI, integrated radius, and Max DAR_2 mm was maintained, while that of SP A1 and the two IOP parameters diminished. The percentage reduction in both IOPuct and bIOP was 16% at 6 months after the operation, but it decreased to 11% at 9 months. The comparison is shown in Figure 3. 

### 3.3. COD and CT

#### 3.3.1. COD

**Figure 4:**
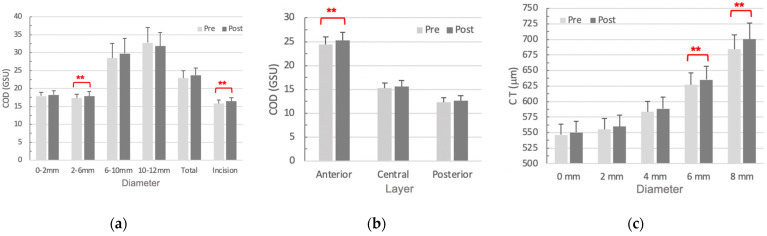
Changes of COD and CT 6 months after a cataract surgery. (**a**) COD of total thickness showed a significant postoperative increase in the 2–6 mm annuli and incision site. (**b**) The anterior 120 μm depth was the only significantly increased layer of COD in 2–6 mm annuli. (**c**) The significance of CT changes remained in the peripheral cornea. Significant differences defined as *p* < 0.05 were highlighted with **. Abbreviation: COD, corneal optical density; GSU, grayscale unit; CT, corneal thickness.

We first analyzed the postoperative COD changes in concentric annuli (Figure 4a). Only the annuli of 2–6 mm diameter showed a significant increase (*p* = 0.045), which was mainly caused by the COD changes of the anterior layer (*p* = 0.039) (Figure 4b). Moreover, we calculated the change of COD at the incisional site, which still increased significantly in 6 months after the surgery (*p* = 0.029) (Figure 4a).

#### 3.3.2. CT

The CT was also analyzed in concentric annuli of diameters 0.0, 2.0, 4.0, 6.0, and 8.0 mm. The central cornea (0–4 mm) recovered to its original thickness within 6 months after cataract surgery, while the peripheral cornea remained swollen. The 6.0- and 8.0–mm annuli significantly thicken when compared with preoperative status (*p* = 0.016 and *p* = 0.015, respectively) (Figure 4c).

### 3.4. Correlation between COD and Biomechanics 

The anterior depth of 2–6 mm annuli is significantly correlated with Max DA (R= −0.500, *p* = 0.049), Max DA_c (R= −0.543, *p* = 0.03), Max DAR_2 mm (R= −0.677, *p* = 0.004), and SP A1 (R = 0.507, *p* = 0.045) (Table 4). The former three exhibited a negative correlation, while the latter showed a positive correlation. However, there was no significant correlation between the total thickness of 2–6 mm annuli and the incision site.

## 4. Discussion

In the current study, we aimed to explore extended biomechanical effects and addressed the unmet issue from shorter observation times in previous research [8,9,10,14].

First, we doubled the postoperative observation time from 3 months to an average of 6 months. The Corvis ST examination showed that the DA_c increased at all phases including A1, A2, HC, and of limit, with accelerating deformative velocity at A1 and A2 (Table 2). The 6-month results were similar to previous 3-month findings [8,10]. Second, we did a subgroup analysis among those with a 9-month follow-up period. The statistical significances were confined in HC and of limit values of the basic Corvis ST parameters (Table 3). A1 and A2 indices had lost significance when compared with short-term results [8,10]. 

Furthermore, the IOP displayed a similar changing pattern. The 6-month results were consistent with prior studies—both IOPuct and bIOP presented with a remarkable decrease (Table 2). Hirasawa et al. illustrated the bIOP dropping percentage of 7.6%, 9.3%, and 10.2% (all *p* < 0.01) one week, one month, and three months after surgery, respectively [9]. Our review showed an approximately 16% reduction (*p* < 0.001) in both IOPuct and bIOP six months following surgery. However, in the 9-month subgroup, the degree of IOP reduction diminished to 11% (Figure 3). Meanwhile, it no longer exhibited significant differences between the pre- and post-operative values (*p* = 0.09 of IOPuct; *p* = 0.12 of bIOP) (Table 3). The changes in IOP showed a diminishing trend after 6 months following the cataract surgery.

The IOP-lowering mechanism of cataract surgery was multifactorial. Lens extraction itself was proven to effectively reduce IOP mainly by changing anatomy, including a deeper anterior chamber, widening Schlemm’s canals, and dislodged zonula [21,22,23]. Thus, cataract surgery was employed as an early treatment option for glaucoma [24]. Damaging the cross-link of collagen fibers induces weakened cornea biomechanics [8,25,26]. Ultrasound energy may be one of the biochemical factors that led to the release of inflammatory cytokines and trabecular meshwork remodeling [27]. Moreover, the resulting higher COD could overestimate the IOP [28]. Therefore, even if the bIOP was corrected for structural factors such as CCT and corneal biomechanics, it still dropped significantly after the cataract surgery, no matter in populations with or without glaucoma [23]. 

The SSI is an updated parameter of the Corvis ST introduced in 2019 to better demonstrate corneal biomechanics with the non-linear feature of the whole corneal behavior [29,30]. It is independent of IOP and positively correlated with age. The value increases with greater stiffness. Aoki et al. first reported a significant SSI reduction in 3- and 6-month post-cataract surgery in open-angle glaucoma (OAG) patients [23]. In agreement with it, the mean SSI in our study also decreased in the 6- and 9-month postoperative period (both *p* < 0.001) (Table 2 and Table 3). The serial SSI changes revealed a less stiff and more deformable cornea in an extended observation period.

Cataract surgery changes not only corneal biomechanics but also IOP, and these two components are closely related. The viscoelastic alterations could make IOP measurement inaccurate; while the IOP reduction also eases the deformation of the cornea [9,10,23]. In the present study, the cataract surgery indeed resulted in a long-lasting impact on the material property of the cornea itself, suggested by the significantly decreased SSI. However, the possible reversibility of IOP alteration may compensate for the performance of other IOP-dependent Corvis ST indices, making the DA, deformative time, and velocity in the A1 and A2 phases not differ significantly from their original values. Although more complete and larger studies are needed, the results indicated that beyond a certain cut-off period, the IOP and biomechanical changes stabilized. Therefore, there is no need to worry too much about continuous deterioration; it might even reverse instead.

The CBI is well-known for the diagnosis of keratoconus [31]. In the studies of cataract surgery, Hirasawa et al. found that the CBI increased in one week postoperatively, but gradually decreased thereafter, even returning to preoperative value in a 3-month follow-up [9]. Our study demonstrated no significant changes in CBI in extended postoperative observation (Table 3). The results suggested a rapid recovery of CBI following cataract surgery. On the other hand, CBI was clinically applied to distinguish keratoconus from healthy eyes. Once the CBI exceeds the calculated cut-off value of 0.5, the cornea is more likely to be keratoconic [32,33]. The biomechanical effects of cataract surgery might be too subtle to make a significant difference in CBI as that of keratoconus.

COD is another notable parameter for monitoring the cornea. COD has better specificity but less sensitivity than CT in reading the corneal alterations [14]. Hsieh et al. revealed increased COD within the full thickness of the central 6 mm annulus one month following cataract surgery [14]. The significance of our extended observation was limited to the anterior 120 μm of 2–6 mm corneal annulus (Figure 4a,b). The incision site remained with a significant increment from one month to nine months after surgery. 

Surgical manipulations, including instruments and phacoemulsification energy, can do direct and indirect harm to the cornea and endothelium [11]. The resultant corneal edema, inflammation, stroma fiber hyperplasia, and fibrosis cause COD, CT, and biomechanics changes [34]. However, the endothelial dysfunction was limited at the cellular level without compromising the pumping activity under smooth cataract surgeries [12,35]. Even stromal fibrosis still has the potential to resolve once the functions of the epithelial basement membrane are fully regenerated [36,37]. Fortunately, the impact was observed predominantly in the periphery, leaving a clear central cornea and minimally affected postoperative VA. 

Our study found that the postoperative variation of anterior COD in 2–6 mm annulus significantly correlated with that of Max DA_c and Max DAR_2 mm (Table 4). The two corneal biomechanics indices marked the limit values of corneal deformation. As a result, we can conclude that COD not only reflects the corneal transparency for light transmittance but also plays a role in echoing the compliance of the cornea. However, the change of COD at the incision site did not correlate with that of any corneal biomechanics indices. It indicated that the incision had little effect on corneal stiffness, even when the localized COD rose. The incision size also made no difference in affecting corneal biomechanics [10]. However, it was noteworthy that Corvis ST measures only the horizontal meridian of the central 8 mm cornea to represent the whole corneal biomechanics [38], which did not include the incision site. We will need more comprehensive measurements of corneal biomechanics when considering the impact of peripheral incisions.

There were a few limitations in the study. First, due to the rapid and favorable recovery from cataract surgery, patients are less likely to attend long-term visits just for postoperative follow-up. This made it hard to conduct long-term observation with numerous participants in clinical practice. The small sample size and still insufficient observation period were the limitations of our study. In addition, there were three patients with both eyes included, which could cause possible selection bias. Second, the biomechanical effects of corneal incision wounds might be underestimated since the Corvis ST had a limited assessment of the peripheral cornea. Third, the mechanism of how COD affects biomechanics is not yet fully explained. Further studies are needed for the evaluation of other contributing factors for corneal biomechanics changes, such as the cushion effect of the anterior chamber. Fourth, the interpretation of IOP reduction will be more complete if comparing patients with and without glaucoma to better identify the contribution of corneal biomechanical changes.

## 5. Conclusions

We conducted an investigation into the extended impact of cataract surgery, concluding that there is a gradual recovery process across corneal biomechanics, COD, and IOP. Given the reversible trend of postoperative changes, cataract surgery is deemed to have no permanent “side effects” on the cornea. However, it is important for physicians to remain vigilant to the transient instability of IOP during the short-term postoperative period to detect abnormalities earlier.

Cataract formation is a natural part of aging that can affect individuals at some point in their lives, making cataract surgery well worth exploring. Investigating the effects of cataract surgery can lead to informed decisions for individuals with various underlying conditions, ultimately improving eye health as they navigate the aging process.

## Figures and Tables

**Table 1:** Demographic data of participants.

	Overall (*N* = 16)
Sex (*N*)	
Male	9 (56.25%)
Female	7 (43.75%)
Laterality (*N*)	
OD	7 (43.8%)
OS	9 (56.3%)
Age (year)	66.3 ± 7.04
VA bare (logMAR)	
Pre	1.45 ± 0.49
Post	0.36 ± 0.19

Abbreviation: *N*, number; OD, oculus dexter; OS, oculus sinister; VA, visual acuity; logMAR, logarithm of the minimum angle of resolution.

**Table 2:** The changes of the Corvis ST parameters before and after cataract surgery.

Corvis ST Parameters *^a^*	Pre	Post	Difference	*p* Value *^b^*
Non-biomechanical				
IOPuct (mmHg)	16.6 ± 4.23	13.9 ± 3.14	−2.69 ± 2.52	**<0.001** *
CCT ((m)	559 ± 37.1	558 ± 35.4	−0.375 ± 16.2	0.93
Basic parameters				
A1 indices				
A1 Time (ms)	7.51 ± 0.556	7.00 ± 0.358	−0.517 ± 0.409	**<0.001** *
A1 Velocity (m/s)	0.141 ± 0.026	0.164 ± 0.021	0.024 ± 0.024	**0.001** *
A1 DA (mm)	0.157 ± 0.011	0.157 ± 0.007	0.001 ± 0.001	0.83
A1 DA_c (mm)	0.109 ± 0.010	0.144 ± 0.006	0.005 ± 0.008	**0.03** *
A1 DL (mm)	2.55 ± 0.147	2.59 ± 0.129	0.044 ± 0.121	0.16
A1 dArcL (mm)	−0.024 ± 0.004	−0.025 ± 0.003	−0.001 ± 0.003	0.29
A1 Darea (mm^2^)	0.220 ± 0.031	0.219 ± 0.029	−0.001 ± 0.031	0.91
A2 indices				
A2 Time (ms)	21.0 ± 0.772	20.8 ± 0.429	−0.165 ± 0.720	0.37
A2 Velocity (m/s)	−0.324 ± 0.072	−0.412 ± 0.052	−0.088 ± 0.086	**<0.001** *
A2 DA (mm)	0.403 ± 0.066	0.434 ± 0.080	0.031 ± 0.079	0.14
A2 DA_c (mm)	0.126 ± 0.020	0.138 ± 0.027	0.012 ± 0.024	**0.07**
A2 DL (mm)	3.27 ± 0.958	3.35 ± 1.13	0.081 ± 0.667	0.64
A2 dArcL (mm)	−0.030 ± 0.008	−0.032 ± 0.010	−0.002 ± 0.0076	0.34
A2 Darea (mm^2^)	0.303 ± 0.083	0.331 ± 0.124	0.029 ± 0.079	0.17
HC indices				
HC Time (ms)	17.1 ± 0.675	16.9 ± 0.884	−0.144 ± 0.960	0.56
HC DA (mm) *^c^*	1.03 ± 0.135	1.17 ± 0.127	0.141 ± 0.101	**<0.001** *
HC DA_c (mm)	0.850 ± 0.148	0.984 ± 0.138	0.134 ± 0.114	**<0.001** *
HC DL (mm)	6.36 ± 0.768	6.76 ± 0.690	0.391 ± 0.547	**0.01** *
HC dArcL (mm)	−0.154 ± 0.042	−0.175 ± 0.038	−0.021 ± 0.039	**0.053**
HC Darea (mm^2^)	3.02 ± 0.758	3.68 ± 0.812	0.663 ± 0.641	**<0.001** *
Limit indices				
WEM (mm)	0.286 ± 0.062	0.309 ± 0.075	0.022 ± 0.080	0.29
WEM time (ms)	21.7 ± 0.916	21.6 ± 0.808	−0.160 ± 1.06	0.55
Max DA (mm) *^c^*	1.03 ± 0.135	1.17 ± 0.127	0.141 ± 0.101	**<0.001** *
Max DA_c (mm)	0.872 ± 0.139	0.997 ± 0.139	0.125 ± 0.112	**<0.001** *
Max DA_c time (ms)	16.1 ± 0.854	16.3 ± 0.984	0.187 ± 1.43	0.61
Max dArcL (mm)	−0.180 ± 0.049	−0.206 ± 0.042	−0.025 ± 0.054	**0.08**
Max ICR (mm^−1^)	0.177 ± 0.016	0.184 ± 0.026	0.008 ± 0.021	0.16
Radius (mm)	7.41 ± 1.14	6.95 ± 1.21	−0.456 ± 0.742	**0.03** *
Peak Distance (mm)	4.72 ± 0.455	5.03 ± 0.418	0.305 ± 0.291	**<0.001** *
Integrated parameters				
bIOP (mmHg)	14.6 ± 3.77	12.2 ± 2.83	−2.39 ± 2.32	**<0.001** *
SSI (mmHg/mm)	1.33 ± 0.240	1.15 ± 0.270	−0.179 ± 0.150	**<0.001** *
PachySlope (a.u.)	37.8 ± 11.5	41.4 ± 17.0	3.66 ± 10.1	0.17
Max DAR_1 mm (a.u.)	1.53 ± 0.039	1.55 ± 0.028	0.011 ± 0.033	0.20
Max DAR_2 mm (a.u.)	4.12 ± 0.379	4.31 ± 0.291	0.191 ± 0.244	**0.007** *
ARTh (mm)	646 ± 165	550 ± 187	−96.7 ± 180	**0.005** *
Integrated Radius (mm)	7.69 ± 0.890	8.16 ± 0.761	0.469 ± 0.634	**0.01** *
SP-A1 (mmHg/mm)	110 ± 26.4	92.7 ± 20.1	−16.8 ± 15.7	**<0.001** *
CBI (a.u.) *^d^*	0.177 ± 0.239	0.299 ± 0.255	0.122 ± 0.256	0.16 *^d^*

*^a^* All variants were shown in mean ± standard deviation; *^b^* Statistical test by Student’s *t*-test; *p* < 0.05 was recognized as a significant difference in statistics (*). *p* < 0.10 was highlighted in bold. *^c^* Max DA = HC DA. *^d^* Statistical test by Wilcoxon signed-rank test; statistical significance was defined as above. Abbreviation: IOP, intraocular pressure; uct, uncorrected; CCT, central corneal thickness; A1, the first applanation; A2, the second applanation; HC, the highest concavity; DA, deformative amplitude; DA_c, deflective amplitude; DL, deflective length; dArcL, the change of arc length within 7 mm corneal center; Darea, deflective area; WEM, whole eye movement; Max, maximal; ICR, inverse concave radius; bIOP, biomechanics-corrected IOP; SSI, stress-strain index; PachySlope (a.u.), the slope of corneal thickness (arbitrary unit); DAR_1 mm (DAR_2 mm), DA ratio between cornea apex and paracentral 1 mm (DAR_2 mm); ARTh, Ambrosio relational thickness at horizontal profile; SP-A1, stiffness parameter at A1, the difference between the strength of the air puff at the corneal surface and the bIOP divided by A1 DA_c; CBI, corneal (or Corvis ST) biomechanical index.

**Table 3:** Subgroup analysis for the extended following period.

Corvis ST Parameters *^a^*	Pre	Post	Difference	*p* Value *^b^*
Non-biomechanical				
IOPuct (mmHg)	18.5 ± 4.15	16.4 ± 1.81	−2.07 ± 2.73	**0.09**
Basic parameters				
A1 indices				
A1 Time	7.66 ± 0.56	7.30 ± 0.23	−0.36 ± 0.40	**0.054**
A1 Velocity	0.13 ± 0.03	0.15 ± 0.02	0.02 ± 0.02	**0.09**
A1 DA_c	0.11 ± 0.01	0.11 ± 0.01	0.00 ± 0.01	0.40
A2 indices				
A2 velocity	−0.35 ± 0.08	−0.38 ± 0.05	−0.04 ± 0.06	0.14
HC indices				
HC DA (= Max DA)	0.98 ± 0.15	1.07 ± 0.10	0.09 ± 0.09	**0.03** *
HC DA_c	0.78 ± 0.16	0.88 ± 0.10	0.10 ± 0.08	**0.02** *
HC DL	6.22 ± 0.97	6.40 ± 0.73	0.18 ± 0.42	0.31
HC Darea	2.70 ± 0.81	3.09 ± 0.56	0.40 ± 0.38	**0.03** *
Limit indices				
Max DA_c	0.81 ± 0.16	0.89 ± 0.10	0.08 ± 0.08	**0.045** *
Radius	7.50 ± 1.37	6.93 ± 1.69	−0.57 ± 0.85	0.12
Peak distanceDistance	4.49 ± 0.51	4.76 ± 0.33	0.27 ± 0.26	**0.03** *
Integrated parameters				
bIOP	16.2 ± 4.32	14.4 ± 2.39	−1.77 ± 2.62	0.12
SSI	1.47 ± 0.25	1.30 ± 0.24	−0.17 ± 0.16	**0.03** *
Max DAR_2 mm	3.85 ± 0.26	4.09 ± 0.25	0.24 ± 0.10	**<0.001** *
ARTh	644 ± 153	542 ± 166	−102 ± 89.61	**0.02** *
Integrated Radius	7.10 ± 0.70	7.78 ± 0.97	0.68 ± 0.61	**0.03** *
SP A1	116 ± 20.9	105 ± 12.1	−11.7 ± 15.7	0.10
CBI *^c^*	0.09 ± 0.07	0.25 ± 0.26	0.15 ± 0.25	0.15 *^c^*

*^a^* All variants were shown in mean  ±  standard deviation; ***^b^*** Statistical test by Student’s *t*-test; *p* < 0.05 was recognized as a significant difference in statistics (*). *p* < 0.10 was highlighted in bold. *^c^* Statistical test by Wilcoxon signed-rank test; statistical significance was defined as above. Abbreviation: IOP, intraocular pressure; uct, uncorrected; A1, the first applanation; DA_c, deflective amplitude; A2, the second applanation; HC, the highest concavity; DA, deformative amplitude; DL, deflective length; Darea, deflective area; Max, maximal; bIOP, biomechanics-corrected IOP; SSI, stress-strain index; DAR_2 mm, DA ratio between cornea apex and paracentral 2 mm; ARTh, Ambrosio relational thickness at horizontal profile; SP-A1, stiffness parameter at A1, the difference between the strength of the air puff at the corneal surface and the bIOP divided by A1 DA_c; CBI, corneal (or Corvis ST) biomechanical index.

**Table 4:** Correlation between the significant parameters of COD and biomechanics.

	2–6 mm Annuli				Incision Site	
	Anterior 120 μm		Total Thickness			
	R *^a^* (%)	*p* Value	R *^a^* (%)	*p* Value	R *^a^* (%)	*p* Value
A1 Time	42.8	**0.098**	34.2	0.195	17.5	0.517
A1 Velocity	−31.2	0.239	−21.0	0.435	−13.3	0.623
A1 DA_c	13.6	0.615	−0.3	0.994	12.2	0.653
A2 Velocity	2.2	0.935	8.8	0.747	7.1	0.795
HC DA_c	−47.4	**0.064**	−39.1	0.134	−7.5	0.783
Max DA	−49.9	**0.049** *	−48.2	**0.059**	−2.4	0.933
Max DA_c	−54.3	**0.03** *	−48.2	**0.059**	24.1	0.369
Peak Distance	−43.5	**0.092**	−31.2	0.239	−33.4	0.208
Radius	−16.5	0.541	−22.0	0.413	7.4	0.785
bIOP	45.6	**0.076**	33.4	0.207	−33.8	0.200
SSI	21.4	0.425	14.3	0.598	−11.6	0.672
Max DAR_2 mm	−67.7	**0.004** *	−36.6	0.165	24.1	0.369
ARTh	3.3	0.901	−22.4	0.404	10.2	0.707
Integrated Radius	−30.6	0.251	−13.8	0.610	2.3	0.934
SP A1	50.7	**0.045** *	40.8	0.116	−20.2	0.453
CBI *^b^*	−33.0	0.211	−3.3	0.905	−18.6	0.491

*^a^* Statistical test by Pearson correlation; *p* < 0.05 was recognized as a significant difference in statistics (*). *p* < 0.10 was highlighted in bold. *^b^* Statical test by Spearman correlation. Abbreviation: COD, corneal optical density; A1, the first applanation; DA_c, deflective amplitude; A2, the second applanation; HC, the highest concavity; Max, maximal; DA, deformative amplitude; bIOP, biomechanics-corrected IOP; SSI, stress-strain index; DAR_2 mm, DA ratio between cornea apex and paracentral 2 mm; ARTh, Ambrosio relational thickness at horizontal profile; SP-A1, stiffness parameter at A1, the difference between the strength of the air puff at the corneal surface and the bIOP divided by A1 DA_c; CBI, corneal (or Corvis ST) biomechanical index.

## Data Availability

The data presented in this study are available on request from the corresponding author. The data are not publicly available due to the protection of patient privacy under IRB regulations.
